# How Psychological Safety Affects Team Performance: Mediating Role of Efficacy and Learning Behavior

**DOI:** 10.3389/fpsyg.2020.01581

**Published:** 2020-07-24

**Authors:** Sehoon Kim, Heesu Lee, Timothy Paul Connerton

**Affiliations:** ^1^Department of Business Administration, Seoul School of Integrated Sciences & Technologies (aSSIST), Seoul, South Korea; ^2^Department of Education, Chung-Ang University, Seoul, South Korea; ^3^Business School Lausanne, Chavannes, Switzerland

**Keywords:** team psychological safety, team learning behavior, team efficacy, team effectiveness, organizational learning, full mediation effect

## Abstract

This article examines the mechanisms that influence team-level performance. It investigates psychological safety, a shared belief that the team is safe for interpersonal risk taking and a causal model mediated by learning behavior and efficacy. This model hypothesizes that psychological safety and efficacy are related, which have been believed to be same-dimension constructs. It also explains the process of how learning behavior affects the team’s efficacy. In a study of 104 field sales and service teams in South Korea, psychological safety did not directly affect team effectiveness. However, when mediated by learning behavior and efficacy, a full-mediation effect was found. The results show (i) that psychological safety is the engine of performance, not the fuel, and (ii) how individuals contribute to group performance under a psychologically safe climate, enhancing team processes. Based on the findings, this article suggests theoretical and methodological implications for future research to maximize teams’ effectiveness.

## Introduction

Teams play a crucial role in highly effective organizations. Teams perform better than individuals (Glassop, 2002), becoming sources for firms’ sustainable competitive advantage. Through horizontal interaction, the knowledge gained by teams contributes to performance on an organizational level (Edmondson, 2012). There is a growing concern about how to improve the performance of teams in organizations. Although a large body of literature has focused on individual motivation over decades, research to advance the understanding of team motivation processes is insufficient (Kozlowski and Bell, 2003; Chen and Kanfer, 2006).

From the literature, physical factors such as team size and task attributes, personal factors such as member competencies and personality, and organizational–environmental factors were studied as antecedents of team effectiveness (TEF) (Cohen and Bailey, 1997; Mathieu et al., 2008). However, organizations are gradually recognizing the value of psychological assets, the importance of synergy among individuals and groups for innovation and growth in highly competitive markets (Donaldson et al., 2011).

The concept of psychological safety appeared half a century ago in the organizational science field, but in recent years, empirical research flourished (Frazier et al., 2017). Previous literature has shown that psychological safety has a direct influence on work performance (Baer and Frese, 2003; Schaubroeck et al., 2011). Besides, more authors insisted that organizational support, safety climate, and performance are unquestionably related, implying that psychological safety might involve benefits that extend its influence on work engagement (Rich et al., 2010; Christian et al., 2011).

Team psychological safety (TPS) is a shared belief that people feel safe about the interpersonal risks that arise concerning their behaviors in a team context (Edmondson, 2018). “Project Aristotle,” which explored over 250 team-level variables, found that successful Google teams have five elements in common: psychological safety, dependability, structure and clarity, meaning, and impact of work (Google, 2015). The findings argue that psychological safety is the most critical factor and a prerequisite to enabling the other four elements. However, surprisingly, despite the importance of that psychological factor, only 47% of employees across the world described that their workplaces are psychologically safe and healthy (Ipsos, 2012).

As Edmondson (2018) pointed out, TPS is the engine of performance, not fuel. Various factors affect the mechanism in the underlying process. What we need to understand is “how” psychological safety leads to team performance. What is necessary for identifying such mechanisms are (i) extended, sustained research at group level and (ii) expansion of the studies in various contexts (e.g., country and culture). Notably, research conducted at the group level is insufficient compared to those conducted at the individual level in psychological safety literature. If related work continues and data accumulate, the theoretical background to examine the incremental validity issue at the group level will be intensified (Frazier et al., 2017).

In many cases, psychological safety has been studied in limited regions (i.e., advanced economies in the west), and now the research context needs to be expanded (Abror, 2017). There is a need to verify the influence of psychological safety on group performance, enhancing its explanatory potential and applicability in the workplace. Additional research is needed to determine what factors mediate the relationship between psychological safety and group effectiveness.

Psychological safety could affect behavioral outcomes such as team’s creativity (Madjar and Ortiz-Walters, 2009), and both individual learning (Carmeli and Gittell, 2009; Carmeli et al., 2009) and team learning (Edmondson, 1999; Wong et al., 2010). Team learning behavior (TLB) is a symbolic variable that affects TEF. TLB is the process by which members interact, acquire knowledge and skills needed for their work, and share information (Argote et al., 1999), and it raises the team process level to generate performance-oriented ideas. When members learn and improve their problem-solving skills, they can create a competitive organization (Dyer and Nobeoka, 2000). Despite the mediating role of learning that has been empirically demonstrated in previous literature, it still needs to be dealt with as a research subject when considering the significance of learning in modern organizations.

Psychological safety has been linked to several attitudinal outcomes as well. Another factor that drives TEF is efficacy. Team efficacy (TE) is a member’s assessment of team ability to perform job-related activities successfully (Walumbwa et al., 2004). Confidence in the team’s abilities affects performance and aligns the members’ activities on the team level (Gibson et al., 2000; Gully et al., 2002). However, few studies have reported the effects of psychological safety to efficacy to present (Abror, 2017). Therefore, there is a theoretical implication to see how efficacy mediates the relationship between psychological safety and performance at the group level. We selected the team’s learning behavior and efficacy as mediating variables to understand the mechanism for creating TEF. Despite the extensive research and empirical support for the critical role of psychological safety, a few unclear questions remain: How does psychological safety affect TEF? How does it affect learning behavior and efficacy? How does learning behavior mediate the overall relationship, and how does it affect the team’s efficacy? Does TE mediate between psychological safety and TEF?

Our aim in this research is to contribute to the team and psychological safety literature in three ways: (i) bring team literature together with related theories by examining psychological safety and learning behavior as determinants of TE; (ii) extend the TEF model and the traditional input–process–output (I–P–O) framework (Hackman, 1987; Cohen and Bailey, 1997) by integrating psychological safety (as contextual input), learning behavior, and efficacy (as process and team traits) that might stimulate TEF; and (iii) embrace TE as a possible mediator between psychological safety and TEF creation.

## Literature Review and Research Model

### TPS

Psychological safety is “a condition in which one feels (a) included, (b) safe to learn, (c) safe to contribute, and (d) safe to challenge the *status quo*, without fear of being embarrassed, marginalized or punished in some way” (Clark, 2019). TPS is a group variable that describes team context. In the last decade, the concept of psychological safety started attracting attention as a primary factor in predicting TEF.

Results from several empirical studies conducted in various regions and countries show that psychological safety plays a vital role in workplace effectiveness (Edmondson and Lei, 2014). The psychological safety of individuals and their teams’ psychological safety are different constructs (Baer and Frese, 2003). The concept was first pioneered by Schein and Bennis (1965) in organizational phenomena and developed by Kahn (1990) as a representative definition of the psychological safety of an individual.

Creating a psychologically safe workplace is different from being undisciplined or being unconditionally generous to any process or outcome (Edmondson, 2012). Two factors—psychological safety and accountability for performance—identify four types of teams. In this regard, the presence of TPS does not necessarily mean that TEF will increase automatically.

Prior research also focused on the relationship between psychological safety and outcomes such as innovation, employee attitudes, creativity, knowledge sharing, voice behaviors, and communication (Newman et al., 2017). Overall, TPS is known to have a positive association with TEF (Schaubroeck et al., 2011; Kessel et al., 2012; Newman et al., 2017).

Extant literature has found positive associations between psychological safety and learning behavior at different levels (Newman et al., 2017). Several pieces of empirical evidence on such relationships were found in previous literature at the team level (Roberto, 2002; Van den Bossche et al., 2006; Stalmeijer et al., 2007; Bstieler and Hemmert, 2010; Ortega et al., 2010; Wong et al., 2010) and individual level.

In addition to this, the relationship between TPS and efficacy should be confirmed. Abror (2017) argues that TPS affects group efficacy. The author criticized Edmondson (1999) for putting TPS and TE on the same level and argued for the need to identify the relationship between the two factors. Recent studies started arguing that TPS may affect group efficacy (May et al., 2004; Roussin et al., 2016; Hernandez and Guarana, 2018). TPS appears to have a significant effect on team behavior and goal orientation and improves performance while affecting a team’s efficacy (Roussin et al., 2016). The following hypotheses arise from the above background.

H1: TPS positively affects TEF.

H2: TPS positively affects TLB.

H3: TPS positively affects TE.

### TLB

Discussions on team learning arose since Argyris (1986) defined organizational learning and discussed it as a sub-element of a learning organization. Edmondson (1999) used the term “team learning behavior” to distinguish the learning process from learning outcomes.

Team learning behavior is defined as gaining and sharing skills, knowledge, and information about work through the interaction of members (Argote et al., 1999), an iterative team process leading to a change (van Offenbeek, 2001). Gibson and Vermeulen (2003) defined TLB as a process of experimentation, reflective communication, and codification. The three elements are interdependent and difficult to replace. Edmondson et al. (2007) divided the perspective on team learning research into three streams.

Previous literature has shown that there is a positive relationship between TLB and TEF. Zellmer-Bruhn and Gibson (2006) identified the factors that influence team learning, team learning’s effects on task performance, and interpersonal relationships. TLB had a positive effect on TE. Van den Bossche et al. (2006) studied how teams build shared beliefs in a collaborative learning environment and found that team learning improves the perceived performance of a team.

Team learning behavior is also known to be positively associated with the team’s efficacy (i.e., van Emmerik et al., 2011). However, further research is needed to verify the direction of the causal relationship between the two variables.

This study views TLB as a process variable and identifies the relationship between TPS and TEF. Also, TLB’s mediating role between TPS and TE would be identified to confirm its value as a useful predictive tool. This section raises the following hypotheses:

H4: TLB positively affects TEF.

H5: TLB positively affects TE.

### TE

TE has a vital role in team research (Rico et al., 2011). As the importance of creating team-based outcomes has grown, TE has attracted the interest of researchers (Day et al., 2009).

Efficacy is the belief that an individual’s ability or competency to perform a particular task will produce a successful outcome (Bandura, 1986, 1997). When expanded into a group level, it becomes group efficacy, which is the belief of group members that they can accomplish a given task. TE is unlikely to be the sum of individual competence and self-esteem (Bandura, 2000).

The concept of TE, together with team resilience and team optimism, is a representative sub-construct of positive organizational behavior (West et al., 2009). It is an essential antecedent predicting group performance (Werner and Lester, 2001; Gully et al., 2002; Chen et al., 2005; Tasa et al., 2007; Porter et al., 2011; Zoogah et al., 2015). The literature supports that efficacy coordinates group processes, such as decision-making and team communication. The level of belief can lead to different outcomes, even under the same conditions. Several empirical works have proved the effect of TE on team performance (Mathieu et al., 2008). In the TE literature, it appears to influence TEF.

As such, we predicted that TE would activate collective processes and impact group performance. Teams that believe they can succeed in a given task can perform better. TE is expected to play an indispensable role in achieving crucial tasks that require enhanced team performance.

H6: TE positively affects TEF.

### TEF

Concepts such as team performance, characteristics, and attitudes of team members define TEF in a comprehensive way (Shen and Chen, 2007). It is difficult to measure or give TEF one single definition. In earlier literature of TEF, the majority of studies defined “effectiveness” as physical outcomes. However, it gradually expanded to the concept of team performance, characteristics, or member attitudes (Shen and Chen, 2007). Lin et al. (2005) insist that researchers should pay attention to various factors simultaneously at the individual and organizational levels to maximize performance.

Rousseau et al. (2006) summarized studies dealing with individual-level variables that improve TEF. As noted, the effectiveness criteria for defining a team’s performance are not limited to the team’s physical output. In addition to productivity, most studies adopted team member satisfaction, attitudes, and perceived outcomes as essential measures. The most widely used are performance and attitude aspects. In this study, TEF is measured by a team’s perception of their performance. Team performance is the result of a dynamic process of member interaction. In-role behavior describes a state in which team members play a supportive role in achieving goals (Williams and Anderson, 1991). Surveys are common ways to measure perceived team performance (Pearce and Sims, 2002; Pearce and Herbik, 2004). In this study, in-role behavior will measure TEF.

### Theoretical Framework and the Moderating Role of TLB and TE

The theoretical foundation can be put on the social cognitive theory (Bandura, 1988). According to the theory, learning is a cognitive process taking place in a social context and could occur purely via observation or instruction, even without direct reinforcement. Also, one’s sense of efficacy can play a crucial role in approaching goals, tasks, and challenges (Luszczynska and Schwarzer, 2005). The theory adequately describes the mechanism of how psychological safety leads to its outcome variables and the relationship between behavioral changes and cognitive beliefs.

Psychological safety at the group level as a model of TEF uses some forms of the input–process–output (I–P–O) model as a theoretical framework. The I–P–O model is an approach that explains the mechanism of team outcome creation. It was Gladstein (1984) and Hackman (1987) who introduced the I–P–O model to explore the mechanism, and Cohen and Bailey (1997) further expanded the model to the TEF model. This framework suits the structural mediation process that involves TLB and TE as process variables for the research. The framework is still valid in many effectiveness research (Liu et al., 2010; Dulebohn and Hoch, 2017; Escribano et al., 2017; Mansikka et al., 2017).

Team learning behavior is known to mediate the relationship between psychological safety and performance (Edmondson, 1999; Li and Yan, 2009; Brueller and Carmeli, 2011; Kostopoulos and Bozionelos, 2011; Hirak et al., 2012; Huang and Jiang, 2012; Li and Tan, 2013; Ortega et al., 2014). Sanner and Bunderson (2013) found in their meta-analysis that TLB was a significant mediator in a large body of literature. In this regard, we try to confirm TLB’s mediating role in the TEF creation mechanism.

Bandura (1988) identified factors that affect efficacy. Social persuasion is encouragement or discouragement from another person. Also, psychological factors alter the level of efficacy. As noted earlier in the literature review, the team’s efficacy affects the performance (Luszczynska and Schwarzer, 2005), and TLB affects TE.

As such, this study investigates the following mediation effects. The hypothesized relationships of the research model (see Figure 2) are as follows:

**FIGURE 1:**
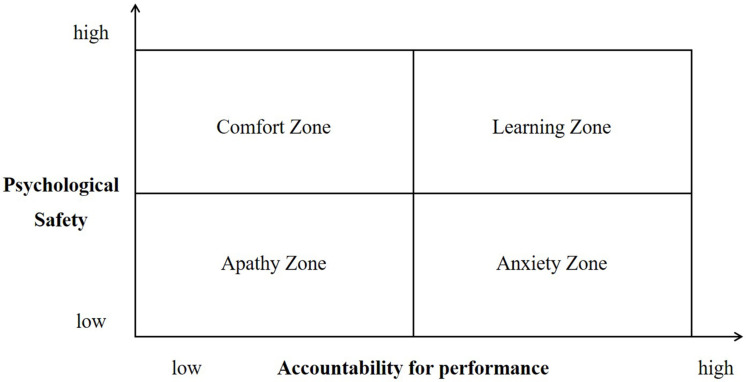
Psychological safety-accountability for performance framework. Source: Edmondson (2012: 174).

**FIGURE 2:**
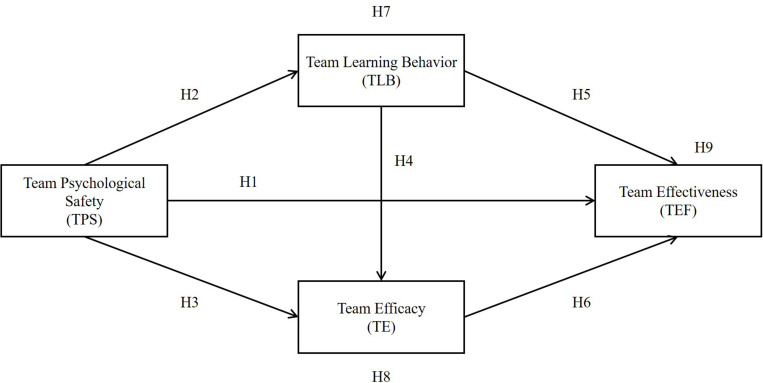
Research model.

H7: TLB mediates the relationship between TPS and TE.

H8: TE mediates the relationship between TPS and TEF.

H9: TLB and TE jointly mediate the relationship between TPS and TEF.

## Research Methodology

### Sample

We collected samples from 16 local sales and service companies located in 98 outlets in South Korea. The survey targeted sales, service, and admin staff working at the front line. Under normal working conditions, field employees providing customer service have to work with a high level of customer orientation, with their service level evaluated continuously. Besides, they are exposed to complaints from dissatisfied customers and feel pressure about their performance, resulting in significant anxiety and stress. Therefore, fieldwork teams were considered appropriate for the research.

A mobile survey was sent to a total of 282 teams and 1,433 employees. Five hundred thirty-six questionnaires were recovered (37%), and 529 valid samples were analyzed. Frequency analysis was performed to examine the distribution of respondents (see Table 1).

**TABLE 1:** Demographic characteristics of the participants.

Attributes		Frequency	(%)	Attributes		Frequency	(%)
Gender	Male	465	87.9	Tenure in team (months)	Under 6	87	16.4
	Female	64	12.1		6∼12	80	15.1
Age	Under 30	120	22.7		13∼24	94	17.8
	30∼35	142	26.8		25∼36	59	11.2
	35∼40	133	25.1		Above 37	209	39.5
	40∼50	132	25.0	Team function	Admin	55	10.4
	Above 50	2	0.4		Sales	236	44.6
Tenure in company (years)	Under 3	222	42.0		Service	238	45.0
	3∼5	132	25.0	Team size	5	149	28.2
	5∼10	121	22.9		5∼7	253	47.8
	10∼15	41	7.8		8∼10	80	15.1
	Above 15	13	2.5		11∼14	22	4.2
Title	Staff	207	39.1		Above 15	25	4.7
	AM	126	23.8	Education	High school	109	20.6
	MGR	96	18.1		Associate degree	200	37.8
	AGM	73	13.8		Bachelor’s degree	206	38.9
	Above GM	27	5.1		Master’s degree	11	2.1
Total		529	100.0		Doctor’s degree	3	0.6
No. of members (mean)		4.5			Member’s age (mean)	31	

Respondents had the following characteristics: gender, 465 male (87.9%) and 64 female (12.1%); age, 30s group highest (26.8% for 30–35 and 25.1% for 35–40), mean = 31; title, staff level highest (39.1%); tenure, under 3 years (42.0%); tenure in the team, 3 years or above (39.5%); team size, less than five members (28.2%), mean = 4.5; team function, admin (10.4%), sales (44.6%), and service (45.0%); and education, bachelor’s degree (38.9%).

### Measurements

#### TPS

Original measurements developed in English were translated to Korean and reviewed by HRD professionals and a group of Ph.D. students to ensure accuracy in the delivery of the meaning. All 27 items adopted a Likert 7-point scale, from 1 = not at all to 7 = to a large extent. TPS consisted of seven questions by Edmondson (1999). Sample items were as follows: “Members are criticized when making a mistake,” “Members often ignore individual’s opinion,” and “Members do not degrade other people’s efforts.”

#### TLB

Team learning behavior adopted nine items from Gibson and Vermeulen (2003). Sample items were as follows: “The team’s ideas and practices are introduced to other teams,” “Members exchange ideas,” and “The team leaves documents about the details of work.”

#### TE

For measuring TE, we adjusted six items by Riggs and Knight (1994). Sample items were as follows: “Members have the best work skills,” “Members have above-average ability,” “The team has excellent performance compared to other teams.”

#### TEF

Team effectiveness was adapted from Williams and Anderson (1991), with the following sample items: “Fulfilling responsibilities given by the organization,” “Achieving the level of task that we expect,” and “Meeting official performance requirements” (see Table 2 and Appendix 1).

**TABLE 2:** Measurement items of the construct.

Variable			No.	Scale	Source
Independent	Team psychological safety		7	Likert 7-point	Edmondson, 1999
Mediator	Team learning behavior	Experimentation	3		Gibson and Vermeulen, 2003
		Reflective communication	3		
		Codification	3		
	Team efficacy		6		Riggs and Knight, 1994
Dependent	Team effectiveness (in-role behavior)		5		Williams and Anderson, 1991

### Analytical Procedure

Process Macro 3.3 was used for the mediated regression model, and Jamovi 1.0.0.0 was used for other analytical procedures, including exploratory factor analysis (EFA) and CFA. First, the demographic distribution was confirmed by frequency analysis. Second, the normality of distribution was tested by descriptive analysis. Third, EFA was carried out to test the variance. Fourth, the confirmatory factor analysis (CFA) secured the validity and reliability of the measurement model. Fifth, we tested the reliability and validity of each team’s value through ICC and *R*_wg_ tests to clear level issues. Sixth, the regression analysis confirmed the relationships between variables. Seventh, statistical significance was confirmed by bootstrap replications. It verified the mediating effects and effect size within the relationships.

## Results

### EFA

There is a possibility of common method bias (CMB) when measuring constructs in the same survey. This issue can lead to the structural underestimation or overestimation of the coefficients (Bido et al., 2017). As criticized by Guide and Ketokivi (2015), researchers should be careful about claiming that the issue is cleared after conducting weak tests, such as Harman’s (1967). In this case, EFA is considered a legitimate statistical procedure to test CMB that supplements the weaknesses of Harman’s, considering both the structural model and the measurement model (Bido et al., 2017). Also, when trying to identify a potential structure or to ensure if the measurements reflected the construct accurately, an additional EFA procedure could be considered, regardless of existing theoretical backgrounds (Fabrigar and Wegener, 2012).

In this study, the maximum likelihood method and oblimin rotation were applied to extract the factors. In the process, variables that did not meet the criteria were removed (factor loadings less than 0.50 and communality less than 0.40). The results of Cronbach’s α confirmed the reliability of measurement instruments (see Table 3).

**TABLE 3:** Result of exploratory factor analysis.

Item	Factor loading				Communality	Cronbach’s α
	
	1	2	3	4		
TPS_1	–	–	–	0.682	0.510	0.793
TPS_2	–	–	–	0.361	0.324	
TPS_3	–	–	–	0.689	0.622	
TPS_5	–	–	–	0.600	0.568	
TLB _ex_1	0.621	–	–	–	0.787	0.922
TLB_ex_2	0.647	–	–	–	0.705	
TLB_ex_3	0.641	–	–	–	0.543	
TLB_com_3	0.519	–	–	–	0.791	
TLB_cod_1	0.758	–	–	–	0.545	
TLB_cod_2	0.847	–	–	–	0.696	
TLB_cod_3	0.810	–	–	–	0.604	
TE_1	–	0.885	–	–	0.787	0.925
TE_2	–	0.755	–	–	0.742	
TE_4	–	0.819	–	–	0.715	
TE_5	–	0.608	–	–	0.704	
TE_6	–	0.622	–	–	0.727	
TEF_1	–	–	0.621	–	0.713	0.916
TEF_3	–	–	0.864	–	0.813	
TEF_4	–	–	0.886	–	0.728	
TEF_5	–	–	0.618	–	0.760	
Eigen value	4.126	3.799	3.107	2.353	–	–
Variance (%)	20.631	18.996	15.533	11.767	66.927	–

*Dimension reduction: Maximum likelihood in oblimin rotation. *n = 529.

It was confirmed that the measurements constituting the four theoretical constructs were grouped into factors without difficulty, and the factor loadings and construct reliability (CR) were also found to be significant. The Bartlett test result showed that the model had a good fit (*P* < 0.001), and the KMO statistics were 0.960, which is also acceptable. TPS question 2 showed a low communality level and was further reviewed for use in the following CFA. Finally, the model was used for CFA after removing three questions from TPS, one from TE, two from TLB, and one from TEF.

### CFA

CFA was conducted to confirm the fit of the measurement model. The criteria for model fit are a chi-square NC (CMIN/*df*) of 5.0 and below, an absolute fitness index (SRMR) below 0.08, an RMSEA below 0.10, and incremental fitness index, TLI, and CFI above 0.90.

The average variance extracted (AVE) value ranged from 0.519 to 0.735, indicating that all variables met the criteria of 0.50 (Bagozzi and Yi, 1988). The internal consistency of Cronbach’s α coefficient was found to be reliable, with all variables above 0.70 or higher (Murphy and Davidshofer, 1988). The standard factor loadings of most items except for one item from TPS and two from TLB were above the recommended level of 0.70 and were significant (*P* < 0.001) (Hair et al., 2017). All the items in CFA were adopted, considering overall AVE (Bagozzi and Yi, 1988). From the above analysis results, the measurement model is acceptable, showing an appropriate level of reliability.

The model fit details are as follows. From the results of χ^2^ = 650 (*P* < 0.001), NC (CMIN/*df*) = 3.963, TLI = 0.933, CFI = 0.942, SRMR = 0.044, and RMSEA = 0.075, no item showed lack in model fit criteria. The reliability analysis results are as shown in Table 4.

**TABLE 4:** Result of confirmatory factor analysis.

Factor	Indicator	Estimate	Std. estimate	SE	*t*-value	CR	AVE	Cronbach’s α
TPS	TPS_1	1.000	0.705	–	–	0.809	0.519	0.793
	TPS_2	0.758	0.568	0.066	11.600***			
	TPS_3	0.956	0.812	0.056	17.000***			
	TPS_5	0.928	0.772	0.060	15.400***			
TLB	TLB_ex_1	1.000	0.899	–	–	0.921	0.627	0.922
	TLB_ex_2	0.894	0.846	0.032	27.600***			
	TLB_ex_3	0.805	0.734	0.038	21.100***			
	TLB_com_3	0.934	0.892	0.030	31.400***			
	TLB_cod_1	0.769	0.682	0.041	18.600***			
	TLB_cod_2	0.921	0.780	0.040	23.300***			
	TLB_cod_3	0.824	0.675	0.045	18.200***			
TE	TE_1	1.000	0.873	–	–	0.927	0.719	0.925
	TE_2	1.033	0.864	0.038	27.300***			
	TE_4	0.953	0.832	0.038	25.400***			
	TE_5	0.894	0.836	0.035	25.500***			
	TE_6	1.130	0.834	0.045	25.300***			
TFE	TEF_1	1.000	0.844	–	–	0.917	0.735	0.916
	TEF_3	1.197	0.879	0.046	26.000***			
	TEF_4	1.168	0.823	0.050	23.300***			
	TEF_5	1.160	0.881	0.045	25.900***			

**Model fit**								

	**χ^2^**	***df***	**NC**	**TLI**	**CFI**	**SRMR**	**RMSEA**	

Criteria	–	–	Under 5.0	Above 0.90	Above 0.90	Under 0.08	Under 0.10	Interval
Result	650	164	3.963	0.933	0.942	0.044	0.075	0.069∼0.081

*n = 529, ***P < 0.001.

### Validity of the Constructs

Convergent validity and discriminant validity were verified to confirm the validity of the construct. Convergent validity is verified by factor loading, CR, and AVE. Convergent validity was confirmed from the measurement model as all the constructs were found to be higher than 0.50 in factor loading, 0.70 in CR, and 0.50 in AVE (see Table 5).

**TABLE 5:** Test of convergent validity.

Item	Factor loading		CR		AVE	
			
Criteria	Above 0.50		Above 0.70		Above 0.50	
			
	Accepted		Accepted		Accepted	
	TPS	0.714	TPS	0.809	TPS	0.519
	TLB	0.787	TLB	0.921	TLB	0.627
	TE	0.848	TE	0.927	TE	0.719
	TEF	0.857	TEF	0.917	TEF	0.735

Discriminant validity means that latent variables are constructs that are independent of each other. If the correlation between factors is relatively high (above 0.80 or 0.85), the researcher can consider a more parsimonious model (Brown, 2015). The results of correlation analysis among the factors are presented (see Table 6).

**TABLE 6:** Correlations between dimensions.

	Mean	SD	1	2	3	4
1. TPS	5.748	1.249	1	–	–	–
2. TLB	5.044	1.177	0.728***	1	–	–
3. TE	5.615	1.109	0.750***	0.769***	1	–
4. TEF	5.669	1.069	0.630***	0.772***	0.857***	1

***P < 0.001.

The *r* ± 2SE method was applied to verify discriminant validity. This method adds and subtracts two standard error range from the correlation values of each factor and checks whether the value includes 1 in the range. The absence of 1 in the calculation range verifies the discriminant validity (Fornell and Larcker, 1981). The *r* ± 2SE range of correlations among all factors did not include 1 (see Table 7).

**TABLE 7:** Test of discriminant validity by *r* ± 2SE method.

	*r*	SE	*r* - (2 × SE)	*r* + (2 × SE)	Including 1
TPS ↔ TE	0.750	0.026	0.698	0.802	N
TPS ↔ TLB	0.728	0.027	0.674	0.782	N
TPS ↔ TEF	0.630	0.033	0.564	0.696	N
TLB ↔ TE	0.769	0.021	0.727	0.811	N
TE ↔ TEF	0.857	0.016	0.826	0.888	N
TLB ↔ TEF	0.772	0.021	0.729	0.815	N

### Level Issue

This study assumes a team-level analysis. Klein et al. (1994) collectively defined three “level issues” that arise in group-level research, which are the level of theory, level of measurement, and level of analysis (Klein et al., 1994). In this study, all the questionnaires measure the team’s view based on a reference-shift model. In the case of using the results of summed or averaged individual responses as a team value, there are two additional requirements as follows.

First, the group members’ responses must be consistent and show homogeneity. Second, the variation or variance between teams should be higher than that within a team. To prove this, Klein and Kozlowski (2000) proposed *R*_wg_ (within-group interrater reliability), intraclass correlation (ICC)(1), and ICC(2). These are the methodologies that support inference for aggregation of individually collected data. This study conducted essential statistical procedures to resolve the level issues before aggregating individual values into a team value.

Checking the consistency and consensus of each rater’s answer to the question solves the problem. ICC is a standard method used for reliability verification in multilevel studies (James, 1982). Reliability refers to the degree of consistency that an individual rater’s evaluation has, and there are two kinds, ICC(1) and ICC(2). Both use analysis of variance to verify data consistency. The usual cutoff level for ICC(1) is 0.20. ICC(2) further supplements ICC(1). It analyzes each group’s composite rating to verify the reliability and is acceptable at 0.60 or higher. The ICC(1) result shows that TEF did not meet the criteria, and TLB and TEF were not acceptable by ICC(2) baseline (see Table 8).

**TABLE 8:** Test of level issue: ICC and *r*_wg_ values.

Factor	ICC(1)	ICC(2)	AVG. *r*_wg_	*F*-value
	**≥0.20**	**≥0.60**	**≥0.50**	
TPS	**0.427**	**0.770**	**0.843**	4.340***
TLB	**0.229**	0.572	**0.870**	2.335***
TE	**0.277**	**0.632**	**0.907**	2.717***
TEF	0.144	0.431	**0.892**	1.757***

*Average team size: 4.5. *n = 471, ***P < 0.001. Bold values indicate meeting standards/cut-off criteria of ICC(1), ICC(2), and *r*_wg_.

*R*_wg_ is an additional verification procedure for the variables which did not meet baseline values. *R*_wg_, also referred to as the within-group agreement index, checks for consistency or reliability of lower-level data (James et al., 1993). Its baseline is 0.70 or higher (James et al., 1993; Klein and Kozlowski, 2000), but variables with *R*_wg_ values higher than 0.50 can be aggregated as a team’s value (James et al., 1993). Finally, a total of 104 team data were analyzed after excluding teams with less than three members and whose *R*_wg_ values did not meet the requirements.

### Hypothesis Test

#### Relationship Between Variables

A process analysis was conducted to verify the effect size on direct and indirect effects simultaneously (Hayes, 2013). By default, a thousand resampling of the percentile bootstrapping method is used to estimate the parameters. The absence of 0 in the 95% confidence interval identifies statistical significance (Preacher et al., 2007; Hayes, 2013). The analysis was carried out based on the Hayes (2013) procedure to verify all the relationships and the direct and indirect effects.

The direct effect of TPS on TE was not significant (H1, β = 0.037). As expected, psychological safety activates team processes but may not direct driver of performance (Edmondson, 2008). TPS had a positive effect on TLB (H2, β = 0.747) and had a significant positive effect on TE (H3, β = 0.596). The result was consistent with previous researches that a sense of safety has a significant impact on team behavior change and performance.

Also, TLB had a positive effect on TE (H4, β = 0.317). The learning process affected the team’s efficacy, which is the team’s emotional response. TLB had a positive effect on TEF (H5, β = 0.193) and was consistent with previous studies’ results that learning improves the quality of task performance.

**FIGURE 3:**
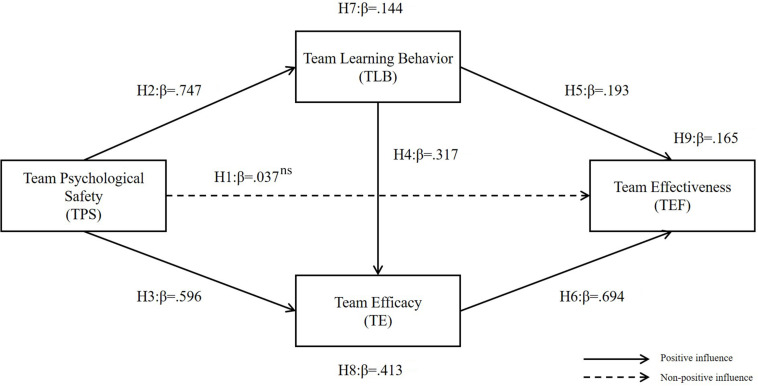
Research model with regression coefficient values. ns: not significant.

Finally, the positive effect on TEF of TE was confirmed (H6, β = 0.694). In summary, TPS did not directly affect effectiveness but had a positive effect on other variables. In the other causal paths, positive causal relationships were identified (see Figure 3 and Table 9).

**TABLE 9:** Result of main effect analysis.

Hypothesis	Path	β	SE	*t*	LLCI	ULCI	Remarks
H1	TPS → TEF	0.037	0.064	0.413	(0.100)	0.153	Rejected
H2	TPS → TLB	0.747	0.060	11.349***	0.563	0.801	Accepted
H3	TPS → TE	0.596	0.063	7.788***	0.367	0.618	Accepted
H4	TLB → TE	0.317	0.069	4.149***	0.150	0.425	Accepted
H5	TLB → TEF	0.193	0.060	2.513*	0.032	0.268	Accepted
H6	TE → TEF	0.694	0.079	7.520***	0.438	0.752	Accepted

*P< 0.05 and ***P< 0.001.

#### Mediating Effect

For the mediating effects to be statistically significant, the indirect effect must show significance in the relationship of the independent variable to the dependent variable. If only the indirect effect is significant in a proposed model, it is a full-mediation effect. In a partial-mediation model, both indirect and direct effects are significant.

The mediating effect of TLB was identified between TPS and TEF (H7, β = 0.144). Also, the mediating role of TE was verified between TPS and TEF (H8, β = 0.413). The effect size was confirmed, and the upper and lower bounds of the 95% confidence interval did not contain 0. TLB and TE showed a double-mediation effect on the relationship between TPS and TEF (H9, β = 0.165).

The total effect of the research model was significant (β = 0.722). The applicability of the research model was supported, and TE was found to have the most substantial indirect effect. The results of the mediation effect analysis are presented (see Table 10).

**TABLE 10:** Total, direct, indirect effect of research model.

Hypothesis	Effect	Path	β	SE	LLCI	ULCI	Remarks
	Total	TPS → TEF	0.722	0.077	0.556	0.861	Full mediation
	Direct	TPS → TEF	0.037	0.064	(0.100)	0.153	–
H7	Indirect 1	TPS → TLB → TEF	0.144	0.070	0.009	0.276	Accepted
H8	Indirect 2	TPS → TE → TEF	0.413	0.074	0.283	0.562	Accepted
H9	Indirect 3	TPS → TLB → TE → TEF	0.165	0.056	0.069	0.286	Accepted

In conclusion, TPS did not directly affect TEF, but TLB and TE indirectly influenced TEF. It also confirmed that TLB contributed to team performance through TE. From the above, the full-mediation and double-mediation effect were found in the research model.

## Discussion

This paper explored how psychological safety influences the team’s effectiveness through learning behavior and efficacy. We applied two mediators in the research design to examine causal relationships. In summary, the research model was found to have a full double-mediation effect. TPS did not have a direct effect on the dependent variable.

First, based on social cognitive theory, we have found the crucial roles of learning behavior and efficacy in connecting psychological safety and TEF. The finding of team learning’s mediation effect is consistent with previous studies (i.e., Kostopoulos and Bozionelos, 2011). Also, the mediating role of TE has been confirmed. To date, little research has been done on the mediating role of TE between psychological safety and TEF. As discussed earlier, psychological factors and climate could alter the level of efficacy. According to social cognitive theory, traits such as the team’s expectations and beliefs could be affected by the psychological factors (environment) and influencing behavior. When members believe that they can complete a given task, the team produces more positive results (e.g., Tasa et al., 2007; Porter et al., 2011).

Second, the results showed that learning behavior positively affects the team’s efficacy. The result was in line with van Emmerik et al. (2011). This finding answers the request of Knapp (2016) for additional research to determine if efficacy is significantly related to learning behavior at the team level. Learning behavior is a process that leads to a shared result and is a link toward change in organizations. If the members recognize excellent communication in the team, they become more involved, and the belief in the team’s ability could be strengthened.

Third, the results did not support one of our hypotheses that psychological safety affects TEF. The research model supported full mediation. This result is consistent with the claims of Edmondson (2012, 2018). Psychological safety is the “engine,” not “fuel” for performance. If individuals are under an atmosphere that highly values their ideas and actions, employees can adapt themselves even to challenging tasks. A team’s psychological safety promotes team learning and consequently increases the team’s effectiveness. Also, the favorable climate promotes the team’s efficacy and contributes to the performance of the team.

## Theoretical Implications

The findings of the study present important contributions to the present knowledge in the domain. First, the research contributes to psychological safety literature by unfolding its little-known relationship with TE, answering the theoretical call from Abror (2017) to examine the relationship between the two constructs. As discussed, we found a significant effect of TPS to TE, confirming the mechanism of how team performance is created through the path.

Today, there is only limited empirical evidence on the effect of psychological safety to efficacy (Abror, 2017). The author criticized Edmondson (1999) for putting the two variables on the same level. Until recently, researchers have insisted that TPS and TE are both psychological factors on the same dimension. Therefore, the causal relationship between the two is rarely experimented. This paper aims to ignite debates on that theoretical discordance in the future based on the full-mediation effect identified.

Recent studies started arguing that psychological safety might affect group efficacy (e.g., Roussin et al., 2016; Hernandez and Guarana, 2018). In the field of education, researchers started reporting the relationship between psychological climate and efficacy. When there is a respectful, collaborative, and trusting school climate (Bryk et al., 2010; Ronfeldt et al., 2013), teachers tend to report higher levels of efficacy and more likely to stay in the profession (Allensworth et al., 2009; Johnson et al., 2012). The research hinted at the theoretical implications and discussions, moving a step forward under the workplace context.

Second, our research contributes to the current literature of TEF by developing and exploring the two different mediating paths, further broadening the boundaries of the studies in human behavior.

The study extends the prevailing framework for TEF (Cohen and Bailey, 1997) by adding empirical data. In the research model, we added a less-proven relationship (i.e., TE as another mediator) to a “psychological safety–team learning–effectiveness” model, further contributing to the applicability and the expandability of the variables as valid predictors in future team studies. To our knowledge, little research has been conducted at a team level, incorporating TPS, TLB, TE, and TEF.

We approached from the aspects of social cognitive theory to explain the TEF creation mechanism that is affected by psychological factors. Prior literature also examined the relationship between psychological safety and other outcomes, integrating theoretical views from social learning theory, social identification theory, social information processing theory, or social exchange theory (Carmeli, 2007; De clercq and Rius, 2007; Schaubroeck et al., 2011; Singh et al., 2013; Chen et al., 2014; Liu et al., 2014; Wang et al., 2018). Our study contributes to building concrete theoretical foundations, enriching various angles available to decipher the complicated phenomena under a team context.

The effect of TLB on TE also presents a new perspective. Previous research has demonstrated that efficacy affects learning behavior (i.e., van Emmerik et al., 2011). However, the studies that reported learning behavior’s effect on TE are limited. This study argues that learning behavior can be a catalyst for the efficacy of teams.

Furthermore, our research answers Frazier et al.’s (2017) call to continue research under the team context. Group-level research is insufficient compared to individual-level studies, and continued research would contribute to the robustness of related theories (Frazier et al., 2017).

Third, the study extended the contexts where psychological safety research takes place. Most of the research was conducted in western countries and advanced economies (Abror, 2017). Moreover, most of the literature dealt with limited work context (e.g., medical, healthcare, and nursing). This research paid attention to frontline sales and service employees in South Korea, broadening boundaries for future empirical work.

## Implications for Practice

The research results may provide several implications for practice. First, the findings point to the vital role of safety climate as a performance enabler in an organization. Top management’s intense pressure can lead to extreme consequences (Edmondson, 2018). Unconditional emphasis on psychological safety is also undesirable. Unrestrained psychological well-being could result in cheating and incompliance with the group’s social constraints (Pearsall and Ellis, 2011). Leaders should pay close attention to establishing an equilibrium that might maximize team performance. Teams can move into a “learning zone” when accountability for performance interacts with psychological safety.

Second, the findings also suggest that energizing the team’s process should be considered for enhanced performance in teams. When a safe environment is ready, members facilitate learning from failures (Hirak et al., 2012), and members’ feedback-seeking behavior and adaptability could be strengthened (Gong and Li, 2019). Therefore, leaders can take a strategy that promotes a psychologically safe climate and stimulates interaction, regardless of external support at a team level. Raising the team’s efficacy would be a superior strategy, too. Regarding the limited resources and authority of team leaders, promoting the team process can be a reliable approach.

Third, the results shed light on the importance of team learning in an organizational context. There are limitations to a top-down approach and centralized training. Learning at a lower level should be stressed as a way of contributing to the firm’s sustainability. Leaders should pay attention to approaches that nurture the dynamic learning process that mediates psychological safety and efficacy, finally leading to performance.

## Limitations and Future Research

In this study, we suggest several limitations as follows. First, as Wang et al. (2018) pointed out, it is still difficult for researchers to infer causal relationships when there is a possible underlying bias from research methodology. In any survey method, some form of bias may be present that leads to the overestimation or underestimation of coefficients or relations (Bido et al., 2017). We collected data from multiple sources based on a single survey followed by a statistical procedure to test the CMB issue. We recommend future researchers of human behavior in business to consider the *ex ante* approach (i.e., the time difference in data collection) so that they can minimize the bias.

Second, longitudinal data collection would provide a stronger theoretical foundation than cross-sectional data. The mediation effect explained by cross-sectional data might not be fully adequate to reveal the hidden structural relationships (Maxwell et al., 2011). Replication of this study based on longitudinal data collection would also be an option for future researchers, re-simulating the findings of the study.

Third, researchers can consider a new line of methodologies and other mediation variables. As Newman et al. (2017) suggested, a qualitative research approach would provide a more holistic and more profound understanding of how psychological safety influences the outcome. With more observational techniques, researches can provide descriptions of a vibrant and dynamic process of a TEF creation. Several factors can influence TEF as a mediator or a moderator. Including little known factors in a research model would provide precious evidence about teams in an era of rapid change.

## Data Availability Statement

The datasets generated for this study are available on request to the corresponding author.

## Ethics Statement

Ethical review and approval was not required for the study on human participants in accordance with the local legislation and institutional requirements. Written informed consent from the patients/participants or patients/participants legal guardian/next of kin was not required to participate in this study in accordance with the national legislation and the institutional requirements.

## Author Contributions

SK devised the research idea, developed the research model, and performed the analytic calculations for the manuscript. HL and TC contributed to the final version of the manuscript and supervised the research. All authors contributed to the article and approved the submitted version.

## Conflict of Interest

The authors declare that the research was conducted in the absence of any commercial or financial relationships that could be construed as a potential conflict of interest.

## Appendix

**TABLE A1:** Questionnaire and descriptive statistics.

Author	Questionnaire	Mean	SD	Skewness	Kurtosis
Edmondson (1999)	Members criticized when making a mistake*	5.254	1.045	0.682	0.536
	Can bring up work problems and awkward stories				
	Members often ignore other people’s opinions*				
	Able to take risks				
	Cannot ask for help from other members*				
	Members do not degrade my efforts				
	My own skills and talents appreciated and utilized				
Gibson and Vermeulen (2003)	Actively propose new ideas for tasks	4.932	1.321	0.500	0.087
	Creating a new way of doing things.				
	Ideas and practices often introduced to other teams				
	Mutual communication	5.535	1.272	1.072	0.977
	Chance to express own opinions				
	Exchange ideas with each other				
	Documents the details of work	4.533	1.409	0.246	0.415
	Records good ideas				
	Records or manages best practice				
	Total	5.000	1.205	0.593	0.236
Riggs and Knight (1994)	Has above-average ability	5.562	1.080	0.781	0.572
	Members have the best work skills				
	Some members can’t do their job properly*				
	Excellent performance compared to other teams				
	Can achieve more than the team’s goal				
	Very efficient				
Williams and Anderson (1991)	Fulfilling responsibilities given by the organization	5.678	1.030	0.702	0.228
	Achieving the level of task that we expect				
	Meeting official performance requirements				
	Doing a key role that can improve team’s evaluation				

**Reverse-coded.*
